# Syncope and Cardiac Arrhythmias Caused by a Paratracheal Mass

**DOI:** 10.31486/toj.23.0032

**Published:** 2023

**Authors:** Sean D. Clayton, Connor Eckholdt, Navdeep Samra, James Cotelingam, Paari Dominic, Vyas R. Rao

**Affiliations:** ^1^ Department of Surgery, Ochsner LSU Health–Shreveport Academic Medical Center, Shreveport, LA; ^2^ Department of Pathology, Ochsner LSU Health–Shreveport Academic Medical Center, Shreveport, LA; ^3^ Department of Cardiology, Ochsner LSU Health–Shreveport Academic Medical Center, Shreveport, LA

**Keywords:** *Calcification–physiologic*, *mass*, *mediastinum*, *syncope*, *syncope–vasovagal*

## Abstract

**Background:** Syncope is a common complaint in clinical practice. The etiologies and mechanisms can be multiple and complex. Syncope caused by a mediastinal mass compressing the vagus nerve is rare.

**Case Report:** We report the case of a patient who presented to the emergency department experiencing recurrent syncope. Imaging revealed a large, calcified mass in the right paratracheal region. After intracranial lesions, cardiac arrhythmias, and orthostatic hypotension were excluded, we suspected that the syncope was related to vagus nerve compression. The patient underwent surgical resection of a mediastinal mass and had complete resolution of syncopal episodes after surgery.

**Conclusion:** This case outcome suggests that recurrent syncope could be the first symptom of an intrathoracic mass.

## INTRODUCTION

Syncope is a common complaint in clinical practice. Many patients have a brief, benign clinical course with spontaneous recovery, with a vasovagal reflex identified as the most common cause of syncope.^1^ Nonetheless, causes of syncope range from benign disorders to life-threatening diseases such as cardiac arrhythmias or structural disease.^2^ Identifying the underlying etiology of syncope can be challenging, especially in complex clinical scenarios with confounding factors. Intrathoracic masses manifesting with recurrent syncope are rare, and the majority of such cases involve the mediastinum with cardiac or pulmonary artery involvement.^3^

We report the case of a patient who presented with recurrent syncope and was found to have an intrathoracic paratracheal mass. Because recurrent syncope caused by a paratracheal mass is poorly described in the literature and is believed to be a rare entity, we present our case to augment the literature regarding this complex pathology.

## CASE REPORT

A 35-year-old female presented for daily syncopal episodes and dizziness. According to the patient, she had undergone an extensive workup by cardiologists in another state prior to her move to Louisiana. She initially presented to an outside cardiologist 3 years prior to the current presentation and reported daily syncopal episodes. The workup included the SEEQ external cardiac monitoring system (Medtronic), cardiac stress test, and echocardiography. Around this time, she had an episode of severe bradycardia and cardiac arrest. She was believed to have symptomatic sinus bradycardia, which led to placement of a permanent pacemaker. Although her symptoms initially resolved, they recurred during the following month.

She reported experiencing a concussion because of her syncopal episodes, without any resulting orthopedic or neurologic injuries. Her medical comorbidities included fibromyalgia, chronic back pain, chronic migraines, endometriosis, and a 4-cm mediastinal mass located superior to the superior vena cava, determined to be histoplasmosis vs endometriosis by a multidisciplinary tumor board review during her previous outside workup. She did not report palpitations, dyspnea on exertion, paroxysmal nocturnal dyspnea, or orthopnea. She did report dizziness during her initial visit and stated that she often “sees stars” and feels a tightness or clenching in the center of her chest just prior to syncopal episodes. However, she reported that syncopal episodes would sometimes occur spontaneously without these prodromal symptoms.

**Figure 1.**
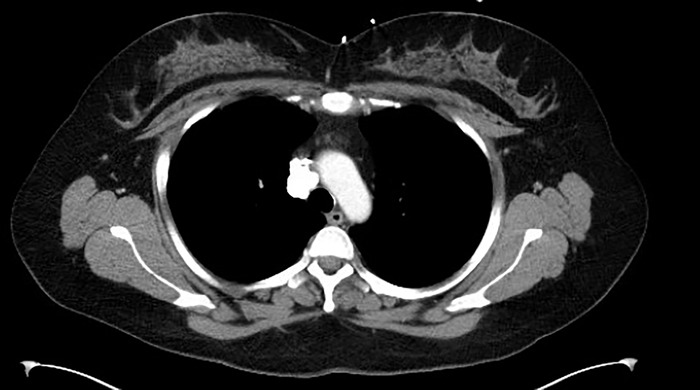
Computed tomography scan shows a large, calcified mass in the right paratracheal area.

Pulmonology consultation raised no concern for superior vena cava obstruction; however, the report suggested that the mass might be compressing the thoracic ganglia and causing autonomic syncope. Since her pacemaker placement, the patient's symptoms had improved, but she continued to have frequent syncopal episodes. Neurology workup with electroencephalography was negative. The patient was referred to otolaryngology to determine if a paradoxical vocal fold motion disorder could be causing vagal episodes. The patient underwent microlaryngoscopy and botulinum injection into the left vocal cord but had no significant improvement in her symptoms. She was referred to cardiothoracic surgery for evaluation of her chest mass; computed tomography showed a heavily calcified mass in the right mediastinum (Figure 1). After a multidisciplinary discussion, the decision was to surgically excise the mass.

**Figure 2.**
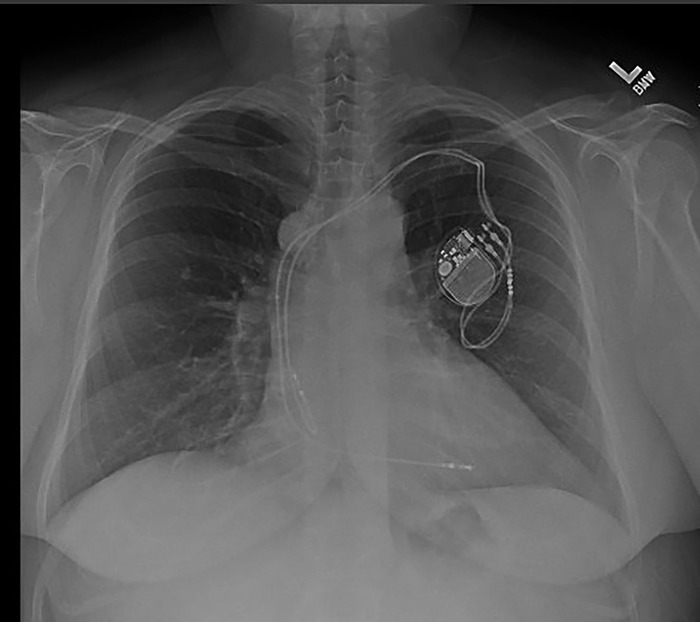
Preoperative chest x-ray shows a calcified mass—the bulge to the left of the aorta near the superior vena cava—in the right paratracheal area.

**Figure 3.**
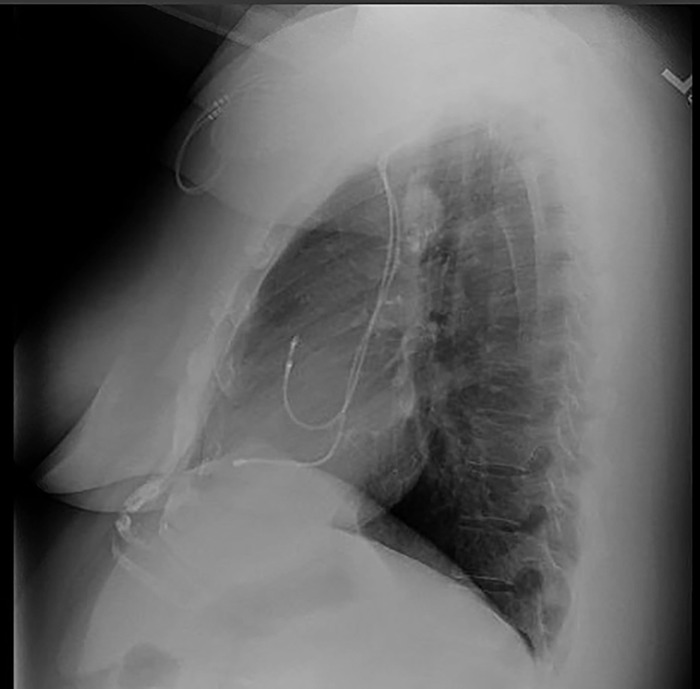
Preoperative chest x-ray lateral view shows the paratracheal mass.

After a discussion of the benefits and risks of the planned procedure, including the possibility that the procedure would fail to improve her symptoms, the patient underwent right thoracotomy and resection of the mass in the right mediastinum. Preoperative chest x-rays showed a calcified mass in the right paratracheal area (Figures 2 and 3). Endotracheal anesthesia was administered, and a double-lumen endotracheal tube was placed. The patient was placed in a left lateral decubitus position. The right side of the chest was prepped and draped. The transverse incision extended from the anterior axillary line to the parascapular line 2 fingerbreadths below the tip of the scapula. The fourth intercostal space was entered, and a golf ball–sized mass was noted in the mediastinum, adherent to the azygos vein. The azygos vein was dissected out of the tumor; however, the medial aspect of the vein was inseparable from the mass. The azygos vein at the junction with the superior vena cava was transected, and the vein on the vena cava side was overrun with a 5-0 PROLENE suture (Ethicon, Inc) in a horizontal mattress technique. The mass was dissected away from the mediastinum and other structures by cautery dissection and then removed. Final pathology identified the mass as a benign calcified lymph node.

**Figure 4.**
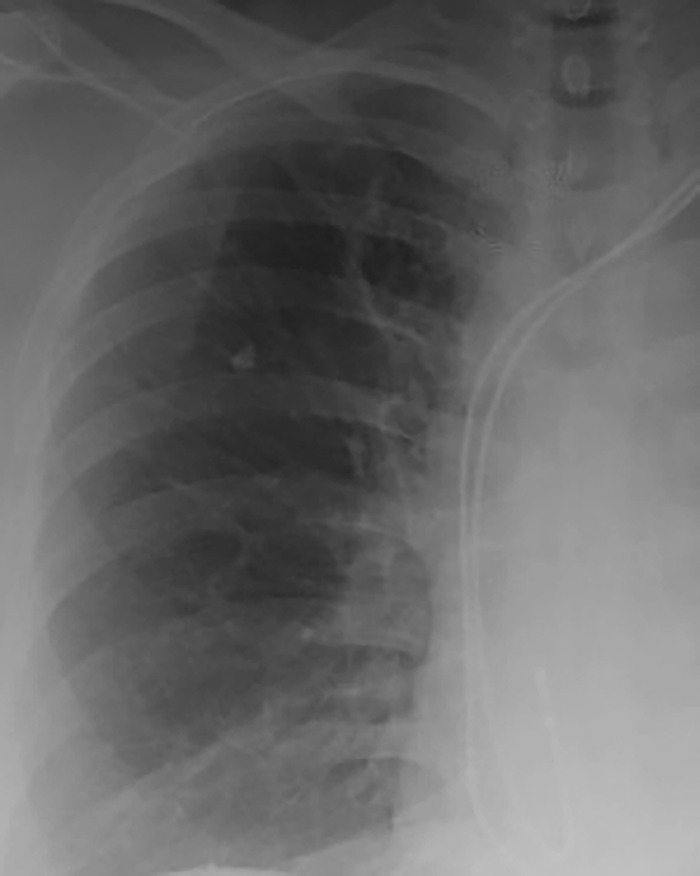
Postoperative chest x-ray shows resolution of the paratracheal mass.

The patient was seen in the cardiothoracic surgery clinic 3 weeks after surgery. Her incisions were well-healed and she reported resolution of her syncopal episodes. Postoperative chest x-ray showed resolution of the paratracheal mass (Figure 4).

## DISCUSSION

Syncope is defined as a transient loss of consciousness with associated loss of postural tone, followed by spontaneous recovery.^1^ The pathophysiology is believed to be secondary to cerebral hypoperfusion, and symptoms such as dizziness, lightheadedness, diaphoresis, nausea, and visual disturbances may precede syncopal episodes.^4^ Syncope accounts for a large number of emergency department visits and hospital admissions yearly in the United States and has varied etiologies, from benign to life-threatening.^5^ Most syncopal events have a benign cause, with vasovagal reflex being the most common etiology. Vasovagal syncope usually has identifiable triggers and a characteristic prodrome, with diagnosis made on history and physical examination.^1,2^ Other causes include orthostatic, neurogenic, postural, situational, and cardiac, with recurrent syncope occurring frequently in elderly individuals with cardiac comorbidities such as arrhythmias or structural abnormalities.^5^

Mediastinal masses are most commonly thymomas, neurogenic tumors, and benign cysts, and they often present with symptoms of cough, chest pain, or shortness of breath.^6^ Syncope is an uncommon symptom of mediastinal masses, with only case reports demonstrating similar presentations. Dúbrava et al reported the case of a mediastinal lymphoma that caused compression of the left atrium and superior vena cava.^7^ Venkatraman et al reported a vasovagal effect similar to our case, in which an intrathoracic neoplasm (B-cell lymphoma) caused significant compromise of the right internal jugular vein and encased the right internal carotid artery, narrowing its caliber and inducing recurrent syncope.^8^ A case reported by Koga et al involved a lung cancer directly invading the vagus nerve.^9^

## CONCLUSION

Our patient presented with a symptomatic mediastinal mass. She had repeated episodes of syncope, followed by complete resolution of symptoms after surgical resection of the mass. This outcome suggests that recurrent syncope could be the first symptom of an intrathoracic neoplasm. Although vasovagal syncope is common, in view of this experience, we suggest that intrathoracic mass remain a differential diagnosis if suggested by the patient's history and physical examination.

## Notes

*This article meets the Accreditation Council for Graduate Medical Education and the American Board of Medical Specialties Maintenance of Certification competencies for Patient Care and Medical Knowledge.*
